# Structure determination reveals the mechanistic basis of mannose-6-P signal generation by the dimeric lysosomal uncovering enzyme NAGPA

**DOI:** 10.1016/j.jbc.2026.113329

**Published:** 2026-07-13

**Authors:** Farha Khan, Chang Sun, Riley Marcinczyk, Amanda Kovach, Balraj Doray, Lin Liu, Huilin Li

**Affiliations:** 1 Department of Structural Biology, Van Andel Institute, Grand Rapids, Michigan, USA; 2 M6P Therapeutics, St Louis, Missouri, USA; 3 Department of Internal Medicine, Washington University School of Medicine, St Louis, Missouri, USA

**Keywords:** Cryo-EM, structural biology, protein glycosylation, mannose-6-phosphate pathway, lysosomal hydrolase targeting

## Abstract

N-acetylglucosamine-1-phosphodiester α-N-acetylglucosaminidase (NAGPA), also known as the uncovering enzyme, catalyzes the final step in mannose-6-phosphate signal generation for lysosomal enzyme trafficking. Despite its central role, the enzyme’s architecture, catalytic mechanism, and the contribution of its C-terminal region remain incompletely defined. Here, we combine solution biophysics, cryo-EM, structural modeling, and a quantitative cell-based assay to characterize human NAGPA. We show that NAGPA forms a noncovalent dimer in solution, resolving prior uncertainty regarding disulfide-linked higher-order assemblies. The structure reveals an elongated dimer composed of two catalytic cores and two C-terminal epidermal growth factor-like stalks, semi-rigid in nature, that likely position the catalytic domains ∼5 nm from the membrane. A structure determined in the presence of the substrate analog GlcNAc-1-phosphate captures GlcNAc and phosphate in the active site, identifying an invariant DGGGS motif that is critical for substrate recognition and enzyme catalysis. Based on these observations, we propose a substrate-assisted S_N_i-like mechanism for cleavage of the glycosidic C-O bond between GlcNAc and mannose-6-phosphate. Functional assays show that the membrane-tethered full-length NAGPA is more active than the isolated catalytic core, and that mutations in the hinge linking the catalytic domain to the C-terminal stalk reduce activity. Together, these findings establish a structural and mechanistic framework for understanding NAGPA function in lysosomal enzyme targeting.

Lysosomes are essential degradative organelles that recycle cellular macromolecules—including proteins, lipids, and nucleic acids—thereby supporting nutrient homeostasis and cellular quality control. Most soluble lysosomal hydrolases are synthesized on endoplasmic reticulum (ER)-bound ribosomes and translocated into the ER lumen, where they acquire N-linked glycans and undergo protein folding, quality control, and sorting events as they traverse the secretory pathway (1). During passage through the Golgi, selected mannose residues on their N-glycans are converted into the mannose-6-phosphate (M6P) sorting signal, a key determinant of lysosomal targeting (2, 3, 4, 5).

M6P generation occurs through a tightly regulated two-step pathway. First, N-acetylglucosamine (GlcNAc)-1-phosphotransferase (PTase) transfers GlcNAc-1-phosphate (GlcNAc-1-P) to the C6 hydroxyl of mannose, generating a phosphodiester intermediate (Man-P-GlcNAc). Second, N-acetylglucosamine-1-phosphodiester α-N-acetylglucosaminidase (NAGPA) removes the terminal GlcNAc to expose the M6P moiety (Fig. 1*A*), thereby earning the designation “uncovering enzyme” (UCE). In the trans-Golgi network (TGN), M6P receptors recognize this signal and sort hydrolases into transport carriers bound for endosomes and ultimately lysosomes, after which the receptors are recycled to the Golgi. Structural studies from our laboratories and others have clarified the upstream steps in this pathway, including N-glycan installation by oligosaccharyltransferase (6, 7) and glycan modification by PTase (8, 9, 10). By contrast, the final uncovering step catalyzed by NAGPA remains less well understood (11).

**Figure 1:**
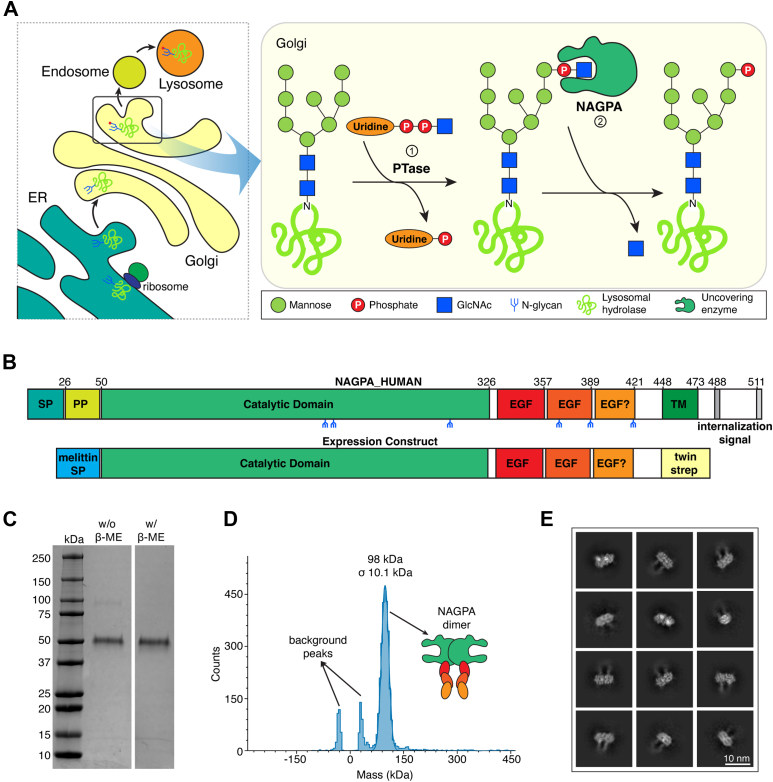
**Biophysical and structural characterization of near full-length human NAGPA.***A*, schematic of mannose-6-phosphate (M6P)-dependent lysosomal hydrolase targeting. Newly synthesized lysosomal hydrolases acquire N-glycans in the ER and traffic through the Golgi, where these glycans are modified in a two-step reaction. First, GlcNAc-1-phosphotransferase (PTase) transfers GlcNAc-1-P from UDP-GlcNAc to N-glycans, generating a phosphodiester intermediate. Second, NAGPA cleaves the GlcNAc cap to expose the M6P tag. M6P-modified hydrolases are subsequently recognized by M6P receptors and delivered to lysosomes. *B*, domain organization of human NAGPA and the soluble recombinant construct used in this study. In the expression construct, the native N-terminal signal sequence is replaced with a melittin signal peptide, and the TM and cytosolic regions are removed, with a C-terminal twin-strep tag appended. Domain boundaries are indicated previously, and predicted N-glycosylation sites are marked below. *C*, SDS-PAGE analysis of purified recombinant NAGPA under nonreducing (-β-ME) and reducing conditions (+β-ME). *D*, mass photometry of purified NAGPA reveals a predominant species at ∼98 kDa (mean ± σ), consistent with a dimer in solution; minor peaks correspond to background noise. *E*, representative 2D class averages showing a dimeric architecture. EGF, epidermal growth factor–like domain; GlcNAc, N-acetylglucosamine; NAGPA, N-acetylglucosamine-1-phosphodiester α-N-acetylglucosaminidase; PP, propeptide; SP, signal peptide; TM, transmembrane helix; β-ME, β-mercaptoethanol.

Human NAGPA (515 amino acid residues) is a type I transmembrane glycoprotein localized to the TGN (12, 13, 14) (Fig. 1*B*). It comprises an N-terminal signal peptide, a pro-peptide, a catalytic core domain, a C-terminal domain, a transmembrane helix, and a cytosolic C-terminal tail. The signal peptide (residues 1–24) directs ER targeting and is cleaved upon translocation. The propeptide (residues 25–49) maintains the enzyme in an inactive state by occupying the catalytic groove (11, 15), and is subsequently removed by the protease furin in the TGN to generate the active enzyme (15). This regulated activation step prevents premature or nonspecific hydrolysis of GlcNAc-containing substrates in the earlier Golgi compartments. The catalytic core (residues 50–325) belongs to a conserved family of phosphodiester glycosidases found from bacteria to humans (16) and carries out hydrolysis of the GlcNAc-1-P-Man linkage to expose the M6P signal (17). Notably, this reaction occurs without recognition of the protein component of lysosomal hydrolases (18). The catalytic core is followed by a cysteine-rich C-terminal stalk composed of epidermal growth factor (EGF)-like domains (19), motifs commonly found in extracellular and cell-surface proteins and thought to play primarily structural roles (20). The C-terminal stalk connects to a transmembrane helix (residues 449–469) that anchors NAGPA in the TGN membrane, followed by a cytosolic tail (470–515) containing sorting motifs (YHPL and NPFKD) required for proper trafficking (21).

Despite its essential role in lysosomal function, several fundamental features of human NAGPA remain unresolved. Early biochemical studies based on native purification identified a ∼120 kDa species under oxidizing conditions, suggesting disulfide-mediated oligomerization, potentially a tetramer (22, 23, 24, 25). Structural information has also remained limited. Small-angle X-ray scattering studies of frog NAGPA suggested that the C-terminal stalk adopts an elongated and potentially flexible conformation (11). Crystal structures of homolog proteins—including the bacterial DUF2233 protein (16) and vertebrate NAGPA constructs from zebrafish, frog, and guinea pig (11)—have provided insight into the conserved catalytic core. However, the overall architecture of human NAGPA, including the arrangement of the C-terminal EGF-like region and its relationship to the membrane, remains unknown. Mechanistic questions also persist: although NAGPA functions as a glycoside hydrolase, the identity and positioning of the general base required for catalysis remain unclear. In addition, genetic studies link NAGPA mutations to non-syndromic stuttering (26, 27), raising the question of how the hinge and C-terminal stalk contribute to enzyme activity and cellular function.

Here, we attempt to address these questions by combining solution biophysics, cryo-EM, structural modeling, and a quantitative cell-based assay. To enable biochemical and structural analysis, we engineered a soluble, secreted form of human NAGPA. We determined structures in both apo and product-bound states and modeled the EGF-like C-stalk within the cryo-EM map. Finally, using a secretion-based assay in NAGPA^−/−^ ExpiCHO cells, we evaluated catalytic and hinge-region variants, establishing how the full-length, membrane-tethered architecture and hinge integrity contribute to uncovering activity in cells.

## Results and discussion

### A soluble near-full-length human NAGPA assembles as a dimer

To investigate the function of human NAGPA and facilitate biochemical characterization, we designed a truncated construct lacking the N-terminal pro-peptide, the transmembrane helix, and the cytosolic tail containing the internalization motifs. This construct was expressed in insect cells as a secreted, soluble enzyme, similar to previous crystallographic studies of other eukaryotic homologs (11).

Purified NAGPA migrated on an SDS-PAGE gel predominantly as a single band at ∼50 kDa under both reducing and oxidizing conditions, indicating minimal inter-domain disulfide bonding. Mass photometry analysis revealed that the enzyme exists almost exclusively as a ∼98 kDa species in solution, in agreement with dimeric assembly, but with no detectable tetrameric population. Consistently, cryo-EM 2D class averages also support a dimeric architecture, showing well-defined densities for the N-terminal catalytic domain and a partially mobile C-terminal stalk.

Together, these results resolve longstanding ambiguity regarding the oligomeric organization of human NAGPA suggested by earlier biochemical studies and argue against the presence of higher-order, disulfide-linked assemblies. However, because these analyses were performed using recombinantly expressed and purified protein, they do not exclude the possibility that NAGPA assembly may be influenced in cells by the membrane environment, local protein concentration, or interactions with other cellular factors. Future live-cell and *in situ* studies will therefore be necessary to establish whether the dimer observed *in vitro* represents the predominant form of NAGPA in its native cellular context.

### Cryo-EM structure of human NAGPA

**Figure 2:**
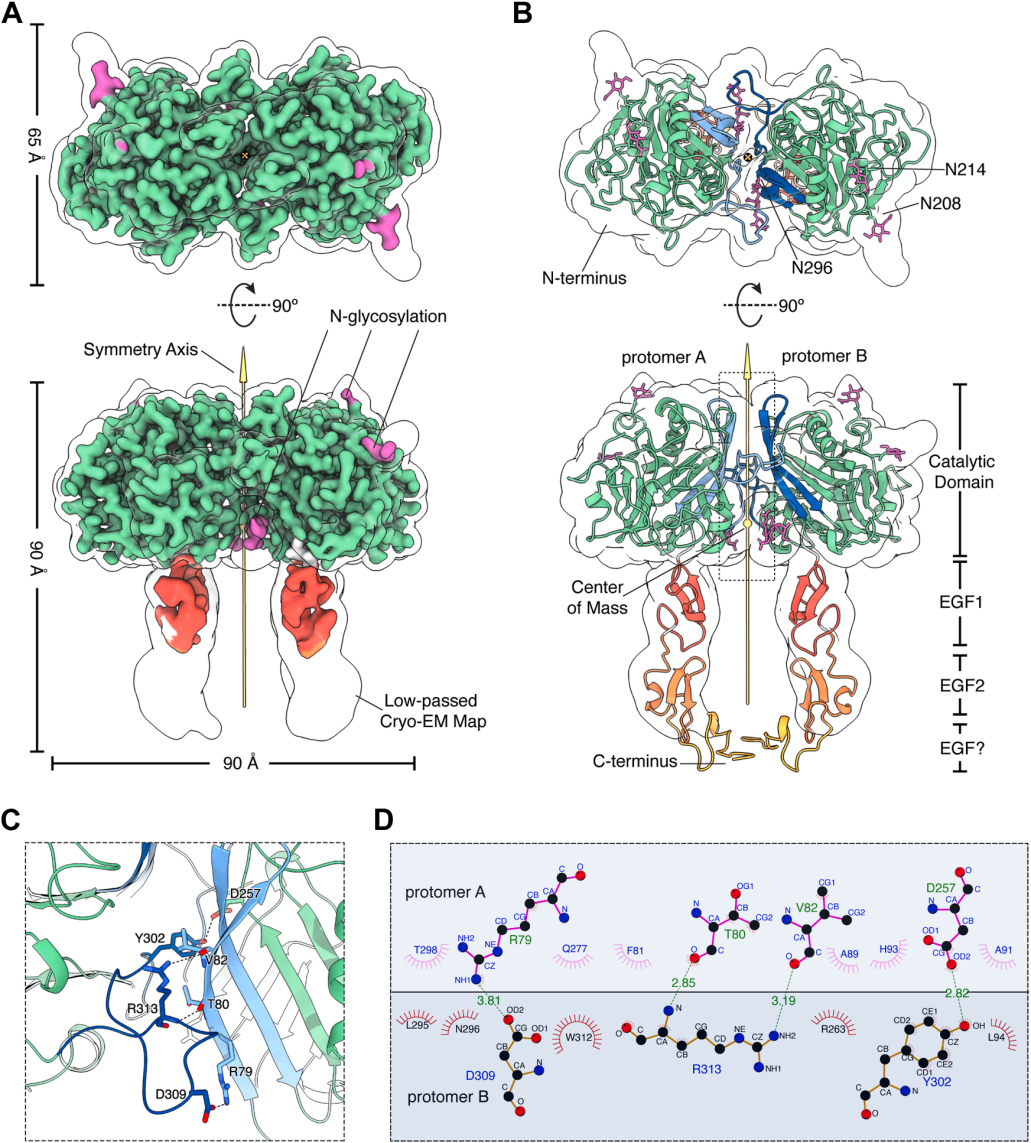
**Cryo-EM structure of human NAGPA reveals a dimeric assembly with peripheral EGF-like domains.***A*, two orthogonal views of the cryo-EM 3D map of the NAGPA dimer. The catalytic domain is shown in *green* with N-glycosylation in *magenta*, while the first EGF-like domain is shown in *red*. A low-pass-filtered map, which better captures the more flexible EGF2 and EGF3 domains, is overlaid as a transparent surface. Approximate dimensions and the dimer symmetry axis are indicated. *B*, atomic model fitted into the cryo-EM map, shown in orientations matching those in panel A. Protomers A and B are labeled and colored according to domain organization. Within the catalytic domain, regions contributing to the dimeric interface are highlighted in *light blue* (protomer A, residues 76–97) and *dark blue* (protomer B, residues 296–317). *C*, enlarged view of a representative inter-protomer interface highlighting residues involved in dimer stabilization. *D*, interaction schematic of the boxed interface shown in panel C, summarizing key inter-protomer contacts, including hydrogen bonds and close-range interactions (distances in Å). EGF, epidermal growth factor; NAGPA, N-acetylglucosamine-1-phosphodiester α-N-acetylglucosaminidase.

We next determined cryo-EM structures of human NAGPA in the as-purified apo state (2.84 Å) and in complex with a truncated substrate analog, GlcNAc-1-P (3.01 Å) (Table S1). In both reconstructions, NAGPA adopts a dimeric architecture with two-fold symmetry relating the protomers (Figs. 2 and S1–S3). The dimer consists of two globular catalytic cores arranged side by side, forming an elongated assembly with a well-defined central interface (Fig. 2, *A* and *B*), consistent with mass photometry measurements (Fig. 1*D*).

The dimer interface buries ∼1267 Å^2^ of solvent-accessible surface area, as calculated by PISA (28), and is stabilized by a combination of hydrophobic packing and hydrogen bonds. The interface is formed primarily by two regions: residues 76 to 97, which contribute two antiparallel β-strands, and residues 296 to 317, which form an extended loop (Fig. 2, *B* and *C*). Within this interface, residues T80 and V82 engage in backbone-mediated hydrogen bonds with R313 from the opposing protomer. Notably, no inter-protomer disulfide bonds are observed between the catalytic domains, suggesting that the previously reported disulfide-linked tetramers likely arose from prolonged, multistep purification of native enzyme preparations (22, 23, 27). Instead, our structural and biochemical analyses support a noncovalent dimer stabilized primarily by extensive surface contacts (Fig. 2, *C* and *D*), which likely explains the persistence of the dimer under oxidizing SDS-PAGE conditions in the absence of heating.

**Figure 3:**
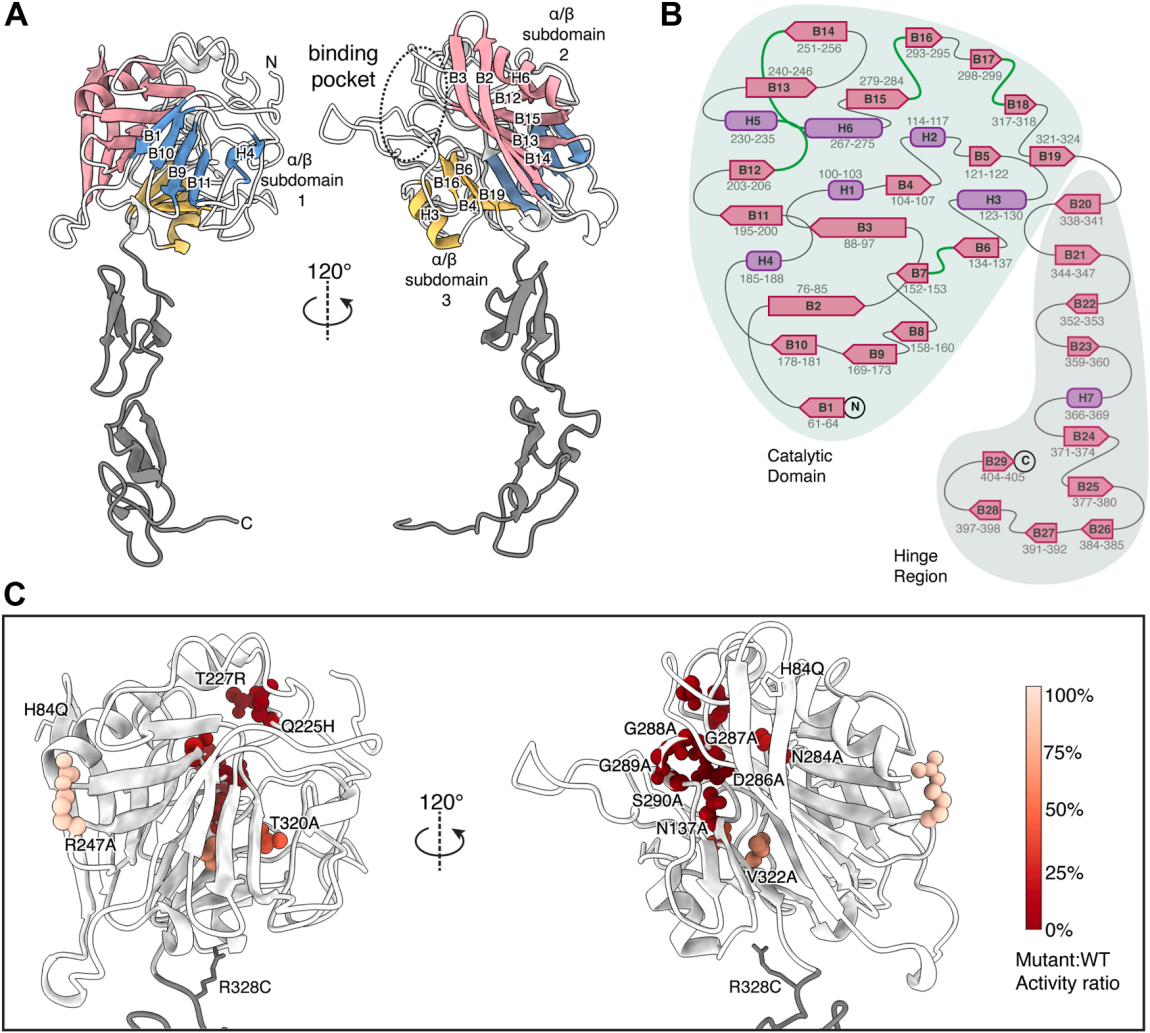
**Secondary structure and topology of the NAGPA protomer.***A*, cartoon representation of a NAGPA protomer shown in two orientations (120° rotation). Secondary-structure elements in the catalytic domain are colored to distinguish the three major α/β subdomains. The C-terminal EGF-like stalk (C-stalk) is shown in *gray*. The N- and C- termini and key secondary structure elements are labeled, and loops forming the substrate binding pocket are highlighted in a *dashed oval*. *B*, topology diagram of the catalytic domain and the C-stalk. β-strands (B1–B29), and α-helices (H1–H7) are labeled with residue ranges. Loops contributing to the catalytic site are depicted in *thick green lines*. The catalytic domain and the stalk are distinguished by shaded backgrounds, with N and C termini indicated to emphasize overall connectivity. *C*, mapping of previously characterized mutations onto the human NAGPA structure. Mutated residues are shown as *spheres* and color-coded according to the extent of activity loss. Two naturally occurring variants associated with persistent stuttering (H84Q and R328C) are labeled and shown as *sticks*. EGF, epidermal growth factor; NAGPA, N-acetylglucosamine-1-phosphodiester α-N-acetylglucosaminidase.

At the level of an individual protomer, human NAGPA is composed predominantly of β-strands interspersed with a limited number of α-helices (Fig. 3, *A* and *B*). The catalytic domain is organized into three α/β subdomains connected by flexible loops (Fig. 3*A*). Each subdomain contains an antiparallel β-sheet capped by a short α-helix, and together the three β-sheets pack into a compact core that defines the overall fold. The junction of the three subdomains is formed by the convergence of five loops, creating a solvent-exposed and moderately flexible pocket on the surface of the catalytic core. Substitution of conserved residues within these loops abolished enzymatic activity (Fig. 3*C*), supporting their role in substrate binding and catalysis. On the opposite side of this junction, a 10-residue loop (residues 65–75) connecting subdomains 1 and 2 contains five proline residues, likely imparting rigidity that stabilizes the catalytic core.

Consistent with sequence-based predictions, the catalytic domain is further stabilized by multiple intramolecular disulfide bonds, including C51–C221, C115–C148, C132–C323, and C307–C314 (19), most of which are conserved across eukaryotic homologs (Fig. S5). With the exception of C132–C323, these disulfide bonds are located in loop regions, where they likely restrict local flexibility and enhance structural stability.

In contrast to prior crystal structures in which the C-terminal stalk was unresolved, our cryo-EM maps reveal additional density extending from the catalytic core consistent with a C-terminal EGF-like stalk (Fig. 2, *A* and *B*). The local resolution permits backbone tracing using CryoAtom (29) for the first EGF domain but decreases substantially for the second and third domains (Figs. S3 and S4). This progressive loss of local resolution likely reflects intrinsic flexibility, as also indicated by 3D variability and 3DFlex analyses (Figs. S1 and S2). To aid interpretation, we generated an AlphaFold3 model (30) and docked it into the low-pass filtered cryo-EM map, yielding a reasonable fit for the EGF1 and EGF2 domains. These analyses suggest that the C-terminal stalk forms a semirigid stalk that would position the catalytic core ∼5 nm from the membrane, elevating the active site above the membrane surface and minimizing steric interference from surrounding glycans within the secretory pathway. Interestingly, an alternatively spliced human NAGPA isoform lacking the putative EGF3 domain has been identified in brain tissue, and both isoforms exhibit comparable enzymatic activity (24), leaving the functional significance of EGF3 unclear.

The cryo-EM maps also provide insight into the glycosylation pattern of the soluble NAGPA construct. Of the six predicted N-glycosylation sites (N-X-S/T motifs) (Fig. 1*B*), well-defined density is observed at N208 and N296, with weaker density observed for N214 (Fig. 2*B*). The reduced density at N214 may reflect lower site occupancy due to steric interference from the adjacent N208 glycan. The remaining three sites are located within the C-terminal EGF-like stalk, which is resolved at lower local resolution, precluding confident assignment. The observed glycan densities are consistent with the modest increase in apparent molecular mass measured by mass photometry (98 kDa *versus* a theoretical mass of 86 kDa; Fig. 1*D*) and provide a structural basis for considering how glycosylation may contribute to NAGPA folding, stability, and function.

### Human NAGPA structure in the presence of a minimal substrate analog GlcNAc-1-P

**Figure 4:**
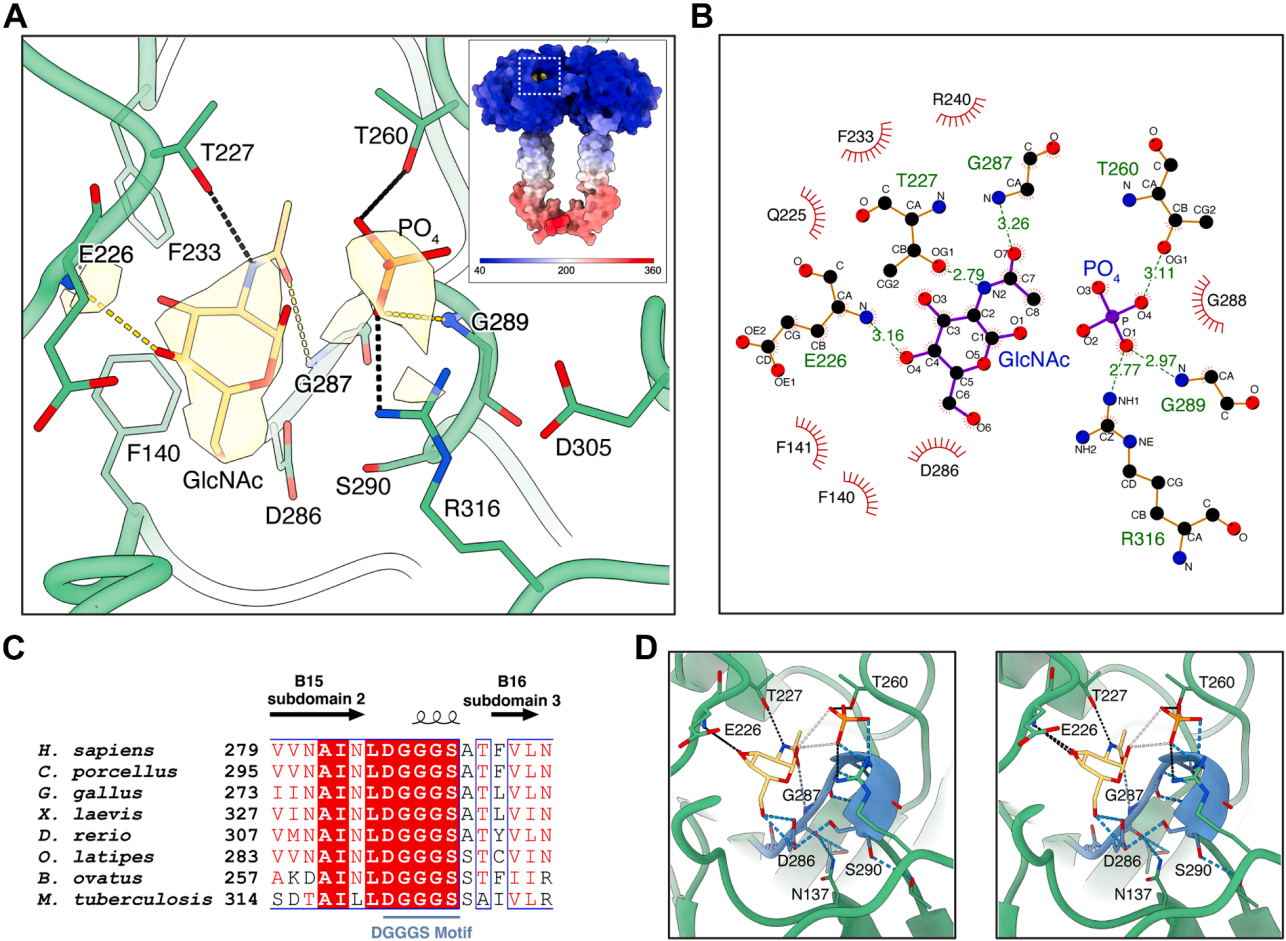
**Structural basis for substrate recognition in the NAGPA active site and a mechanistic model for GlcNAc cap removal.***A*, close-up view of the NAGPA active site in the product-bound state, with GlcNAc and phosphate modeled into the cryo-EM density. Key residues lining the binding pocket are labeled. Inset shows the location of the active site within the dimeric assembly. Cryo-EM density of the bound ligands is shown in *yellow*, and the protein surface is colored by B-factors (Å^2^) of the modeled atoms using the *blue-white-red palette*. *B*, LigPlot summary of interactions between the bound ligands and active-site residues, highlighting hydrogen bonds/polar contacts and hydrophobic interactions. Distances are indicated in Å. *C*, multiple sequence alignment of NAGPA homologs from vertebrates and selected bacterial species identifies a highly conserved loop motif, designated here as the DGGGS motif. *D*, stereo view of the hydrogen-bonding network centered on the DGGGS motif (*blue*) and the bound products. *White dashed lines* denote ligand–ligand hydrogen bonds, *black dashed lines* indicate interactions between ligands and residues outside the DGGGS motif, and *blue dashed lines* represent hydrogen bonds involving DGGGS motif residues, including both ligand–protein and protein–protein interactions. GlcNAc, N-acetylglucosamine; NAGPA, N-acetylglucosamine-1-phosphodiester α-N-acetylglucosaminidase.

To investigate the structural basis of catalysis, purified NAGPA was incubated with a minimal substrate analog, GlcNAc-1-P, for 1 h prior to grid preparation. Although the physiological substrate is a GlcNAc-phosphodiester attached to glycoproteins, previous biochemical studies (16, 17) have shown that truncated sugar–phosphate analogs such as GlcNAc-1-P are turned over with reduced but measurable activity, making them useful probes of the catalytic pocket. Consistent with this rationale, the ligand-incubated cryo-EM reconstruction reveals well-defined density within the active site that is best interpreted as cleaved GlcNAc and a free phosphate ion, capturing a product-like state following glycosidic bond hydrolysis (Fig. 4, *A* and *B*).

The active site is solvent-exposed and formed by a conserved set of residues that coordinate both the sugar and phosphate products (Figs. 4 and S5). In this product-like state, the GlcNAc moiety is stabilized by a hydrogen-bonding network involving side chains of E226 and T227, as well as the backbone amide of G287. Notably, two of these interactions engage the N-acetyl group, providing a structural basis for the enzyme’s selectivity toward GlcNAc. In addition, the catalytic residue D286 (11), although not explicitly captured in the LigPlot analysis, is positioned to form hydrogen bonds with the sugar and anchor it at the base of the pocket (Fig. 4*D*).

The phosphate group is coordinated in a distinct subsite through electrostatic interaction with R316 and hydrogen bonds with the side chain of T260 and the backbone amide of G289 (Fig. 4, *A* and *B*). Together, these interactions define a composite binding pocket that accommodates the sugar and phosphate moieties in spatially separated but adjacent subsites.

**Figure 5:**
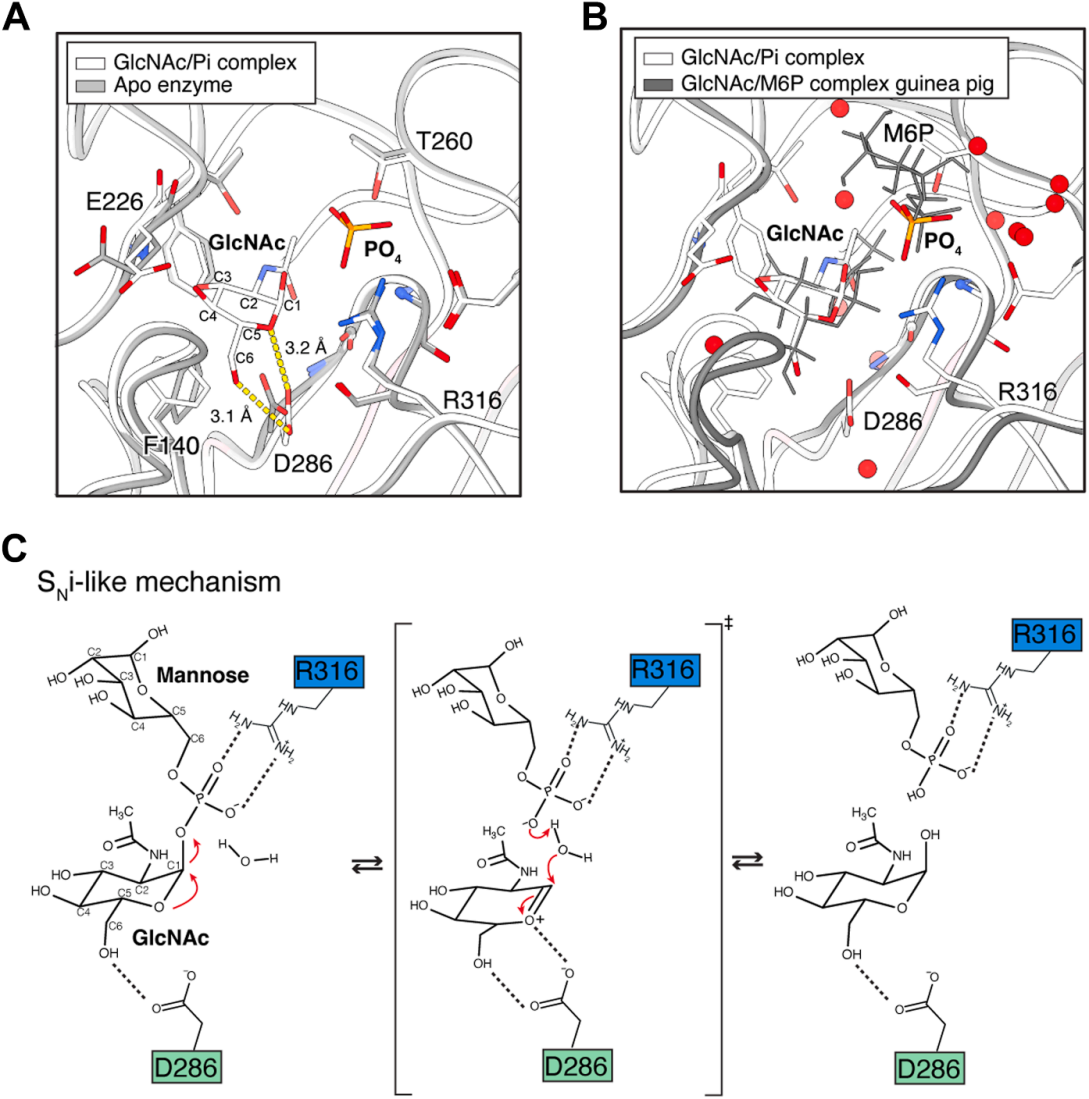
**Ligand binding and proposed reaction model.***A*, structural superposition of apo NAGPA and the GlcNAc/phosphate-bound structure, highlighting local conformational changes within the ligand-binding pocket upon product binding. *B*, structural overlay of the cryo-EM product-bound structure with a previously reported guinea pig NAGPA structure (PDB: 6PKU) containing GlcNAc and M6P, demonstrating conserved features of ligand recognition. Water molecules resolved in the crystal structure are shown as *red spheres*. *C*, proposed substrate-assisted S_N_i-like reaction mechanism for NAGPA. The substrate is positioned in the active site in part through hydrogen bonding between D286 and the C6 hydroxyl of GlcNAc. Cleavage proceeds *via* an oxocarbenium-like intermediate as the phosphate-containing group departs, followed by water attack at the anomeric center to yield the α-linked GlcNAc product. GlcNAc, N-acetylglucosamine; M6P, mannose-6-phosphate; NAGPA, N-acetylglucosamine-1-phosphodiester α-N-acetylglucosaminidase; PDB, Protein Data Bank.

Comparison of ligand-bound and apo structures reveals only localized conformational changes within the catalytic pocket, involving residues such as F140 and D286 at the base, and E226, T260, and R316 along the side walls (Fig. 5*A*). These observations suggest that, following proteolytic removal of the inhibitory propeptide, the active site is largely preorganized to accommodate substrate binding and catalysis.

### An invariant DGGGS motif for sugar-phosphate recognition

Sequence analysis of NAGPA orthologs reveals moderate conservation among eukaryotes (50–85% identity) but markedly lower similarity between human and bacterial enzymes (<20%; Fig. S5). Despite this divergence, one short segment in the human enzyme (286-DGGGS-290) is strictly invariant across evolution (Fig. 4*C*), suggesting a fundamental functional role.

Our structural analysis shows that this motif serves as a key determinant for recognition of the GlcNAc-1-P moiety. The DGGGS motif forms a loop connecting β-strands from α/β subdomains 2 and 3 (Figs. 3 and 4*D*), positioning it within a well-defined structural framework. Within the motif, D286 forms a hydrogen bond with S290, which helps orient the side chain of D286 for productive interaction with the sugar moiety. The three consecutive glycine residues permit a tight backbone turn while minimizing steric constraints, thereby creating a cavity that accommodates the phosphate group through backbone amide hydrogen bonds and favorable local electrostatics. The loop conformation is further stabilized by an internal hydrogen bond between the carbonyl of G287 and the amide of S290, along with additional contacts to surrounding structural elements (Fig. 4*D*). At the same time, the glycine-rich backbone likely retains sufficient flexibility to accommodate subtle positional changes of the phosphate group during catalysis.

Consistent with its functional importance, mutations within this motif severely impair enzyme behavior: substitutions such as G287A and G288A disrupt ER exit, whereas D286A, G288A, G289A, and S290A abolish catalytic activity altogether (16). Notably, the DGGGS motif bears conceptual resemblance to the Walker A motif (GXXXXGK(T/S)) found in AAA+ ATPases (31), which also uses a glycine-rich loop to engage phosphate groups. In both cases, a flexible, glycine-rich backbone—flanked by defined secondary structural elements—enables precise positioning of phosphate-binding interactions.

Together, these observations indicate that the DGGGS motif is structurally and functionally optimized to coordinate the sugar-phosphate group while supporting catalysis, providing a clear rationale for its strict evolutionary conservation among phosphodiester glycosidases.

### Proposed reaction mechanism

NAGPA was initially categorized as a phosphoric diester hydrolase (EC 3.1.4) (32). However, isotope-labeling experiments demonstrated that the enzyme cleaves a glycosidic (C–O) bond rather than a phosphoester (P–O) bond, leading to its reclassification as an acetylglucosaminidase (17). Furthermore, biochemical (13, 22, 23) and proton NMR (11) studies established that the anomeric carbon retains the α-configuration in both substrate and product, indicating that NAGPA operates *via* an α-retaining reaction mechanism.

We find that substrate binding only induced very localized changes in the active site of NAGPA (Fig. 5*A*). Our human enzyme structure aligns closely with the previously reported guinea pig NAGPA crystal structure determined in the presence of GlcNAc and M6P (11), with largely overlapping positions of the GlcNAc and phosphate groups (Fig. 5*B*), suggesting a conserved reaction mechanism. However, a double-displacement mechanism was previously proposed to explain this stereochemical outcome, in which D286 acts as a nucleophile to form a covalent glycosyl–enzyme intermediate, followed by hydrolysis by a water molecule (11). Our cryo-EM structure does not support such a role for D286. Instead, D286 is positioned closer to the C6 hydroxyl (O6) of the GlcNAc moiety and forms hydrogen bonds, rather than being oriented towards the anomeric C1 carbon.

In parallel, accumulating evidence from studies of α-retaining glycosyltransferases (33)—including trehalose-6-phosphate synthase (34), lipopolysaccharyl-α-1,4-galactosyltransferase C (35), and Notch-modifying xylosyltransferase (36)—support a front-face, S_N_i-like mechanism rather than a classic double-displacement pathway. Together, these structural and mechanistic considerations prompted us to propose an alternative reaction model for NAGPA.

We propose that NAGPA operates through an S_N_i-like retaining mechanism (Fig. 5*C*). In this model, the catalytic pocket—organized around D286 and R316—is configured to promote cleavage of the glycosidic C–O bond in GlcNA-1-P-Man. Notably, the phosphate group constitutes an intrinsically favorable leaving group compared to the sugar moiety in canonical glycosidase substrates or amino acid residues in protease substrates. Cleavage of the glycosidic bond generates a transient oxocarbenium-like intermediate, which is stabilized by D286, while the departing phosphate group is stabilized through electrostatic interaction with R316.

We further propose that the departing phosphate group acts as a general base to activate a nearby water molecule, which then attacks the anomeric carbon from the same face, resulting in retention of α stereochemistry. In this model, glycosidic bond cleavage and water activation are tightly coupled. Although the catalytic water is not resolved in our structure—consistent with a product-bound state following hydrolysis—the solvent accessibility of the active site suggests that it is positioned above the sugar moiety, where it remains proximal to the departing phosphate group and connected to the bulk solvent. Supporting this placement, the guinea pig structure contains an ordered water molecule above the GlcNAc moiety (11).

Overall, this mechanism represents a substrate-assisted S_N_i-like retaining hydrolysis, in which the phosphate group of the substrate—rather than an enzyme residue—functions in activating the nucleophilic water, while D286 and R316 facilitate bond breakage and charge stabilization. This model is consistent with prior mutagenesis studies showing that substitutions at D286 and R316 abolish enzyme activity (11). Further studies aimed at capturing reaction intermediates will be required to test this mechanism directly.

### Cell-based functional assay of NAGPA mutants

Previous genetic studies have identified NAGPA variants associated with persistent developmental stuttering, including two missense mutations, H84Q and R328C, and a frameshift mutation that removes the final three residues and appends 113 additional amino acids (26). Among these, R328C maps to the C-terminal end of the catalytic domain near the base of the hinge region. In our structure, R328 forms a hydrogen-bonding interaction with H325, suggesting that this region may contribute to the structural and functional coupling between the catalytic core and the C-terminal stalk. This observation raised the possibility that, in addition to the previously proposed effects of R328C on protein folding or stability (27), disruption of hinge-region interactions could directly influence NAGPA activity in cells.

**Figure 6:**
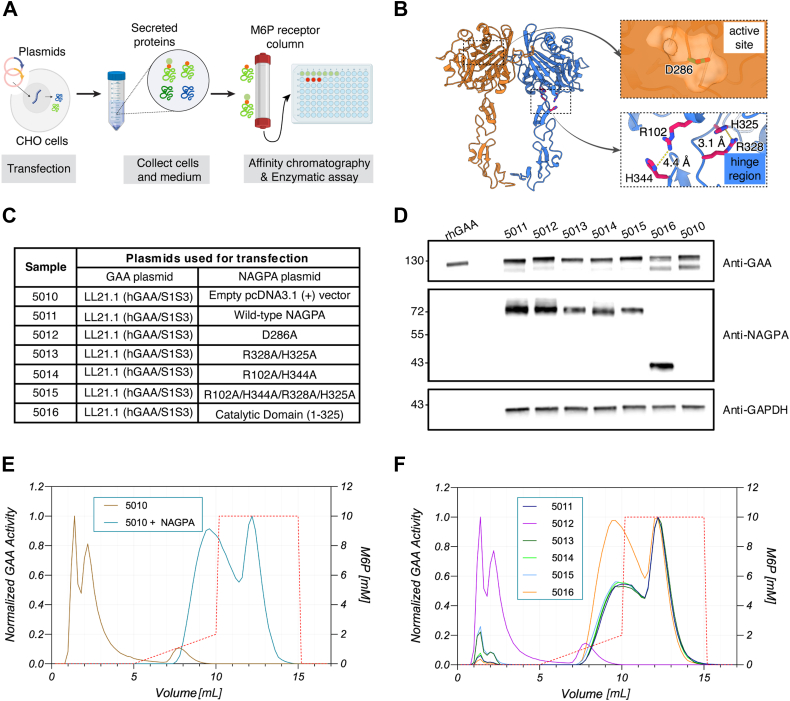
**Cell-based analysis of NAGPA function reveals contributions from the catalytic site and hinge region.***A*, schematic of the cell-based assay. CHO cells were cotransfected with plasmids encoding hGAA and the indicated NAGPA constructs. Conditioned medium containing secreted proteins was collected and applied to a CI-MPR affinity column to enrich M6P-containing hGAA, followed by enzymatic activity measurements across all fractions. *B*, structural context of the NAGPA mutations examined. The *upper inset* highlights the catalytic residue D286 within the active site. The *lower inset* shows a cluster of hinge-region residues, including inter-residue distances that guided the design of hinge-perturbing mutants. *C*, summary of transfection conditions for samples 5010 to 5016, including empty vector control, WT NAGPA, the catalytic mutant D286A, hinge-region double mutants, the quadruple mutant, and the catalytic-domain construct. *D*, immunoblot analysis of cell lysates confirming expression of hGAA and the indicated NAGPA constructs. GAPDH serves as a loading control. *E*, normalized CI-MPR affinity chromatograms showing M6P uncovering of secreted hGAA in the absence of cotransfected NAGPA (sample 5010) or after addition of recombinant NAGPA. hGAA activity is shown as *solid lines*, and the M6P elution gradient as a *red dashed line*. *F*, normalized CI-MPR affinity chromatograms showing M6P uncovering of secreted hGAA coexpressed with WT or mutant NAGPA constructs. In panel E and F, the chromatograms were averaged across three biological replicates. CHO, Chinese hamster ovary; CI-MPR, cation-independent mannose 6-phosphate receptor; hGAA, human acid α-glucosidase; M6P, mannose-6-phosphate; NAGPA, N-acetylglucosamine-1-phosphodiester α-N-acetylglucosaminidase.

To assess how hinge-region integrity contributes to NAGPA function in cells, we developed a secretion-based assay that converts NAGPA-dependent “uncovering” activity into a quantitative readout of cation-independent mannose 6-phosphate receptor (CI-MPR) binding of a secreted reporter (Fig. 6). In this system, *NAGPA*^−/−^ CHO cells are cotransfected with plasmids encoding human acid α-glucosidase (hGAA) and an engineered phosphotransferase (S1S3 PTase) (10, 37)) to increase the production of GlcNAc-capped glycans (step 1 in Fig. 1*A*). NAGPA variants are then coexpressed to catalyze the uncovering reaction and generate M6P (step 2 in Fig. 1*A*). Because hGAA is overexpressed, a fraction of processed enzyme is secreted into the medium, enabling functional analysis without cell lysis. Conditioned media are applied to a CI-MPR affinity column to separate species bearing high-affinity M6P from those retaining the GlcNAc cap, followed by quantitative comparison of elution profiles based on hGAA activity. This assay exploits the strong preference of CI-MPR for phosphomonoesters over phosphodiester-capped glycans (38).

We first established the baseline phenotype of hGAA secreted from *NAGPA*^−/−^ cells in the absence of NAGPA activity. Under these conditions, essentially none of the secreted hGAA (<1%) displayed strong CI-MPR binding: most material failed to bind the column, and only a minor fraction (∼17%) exhibited weak binding and eluted at 1 mM M6P (Fig. 6*E*). Treating the same conditioned medium with purified soluble NAGPA restored CI-MPR binding, with ∼49% of hGAA recovered in the strongly bound fraction and the remainder showing weaker binding (Fig. 6*E*). These results establish the 10 mM M6P-eluted fraction, corresponding to high-affinity CI-MPR-bound hGAA, as a sensitive quantitative readout of NAGPA activity in this cellular assay.

We next used this assay to examine the contribution of the C-terminal stalk, which included the EGF-like domains and the membrane-tethering helix, to NAGPA function in cells. Guided by the structural insights and disease-associated substitutions mapped to the hinge (*e*.*g*., R328C), we generated two hinge-region double mutants (R328A/H325A and R102A/H344A), a quadruple mutant (R102A/H344A/R328A/H325A), a soluble catalytic-domain construct (residues 1–325; lacking both the membrane-tethering segment and the C-terminal EGF-like stalk), and a catalytic-site inactive control (D286A) (Fig. 6, *B* and *C*). Immunoblotting confirmed robust expression of hGAA and all NAGPA constructs in cell lysates, and there were detectable levels of secreted hGAA in conditioned media across all conditions (Figs. 6*D* and S6), indicating that differences in CI-MPR binding primarily reflect functional effects rather than expression variability.

CI-MPR affinity chromatography showed that the catalytic-site mutant D286A abolished activity, yielding an elution profile indistinguishable from the NAGPA-null condition, consistent with the essential role of D286 in catalysis (Fig. 6*F*). The soluble catalytic-domain construct partially restored CI-MPR binding of hGAA but remained less active than full-length WT NAGPA which shows very similar profile as the covered hGAA incubated with exogenous recombinant UCE enzyme. This partial rescue indicates that the catalytic core alone retains uncovering activity, whereas the full-length, membrane-tethered architecture confers an additional functional advantage in cells. We propose that the transmembrane domain and C-terminal sorting motif spatially confine NAGPA to the Golgi apparatus, increasing the frequency of productive encounters with transiting glycoprotein substrates.

In contrast, the hinge-region mutants R328A/H325A and R102A/H344A, as well as the quadruple mutant, retained substantial activity but exhibited intermediate reductions in the strongly CI-MPR-bound fraction relative to WT (Fig. 6*F*). Among these, the quadruple mutant produced the largest defect. Although the mechanistic basis for this effect remains unclear, these results suggest that interactions within the hinge region contribute to the functional coupling between the catalytic core and the C-terminal stalk. While speculative, we propose that the hinge region acts as a flexible pivot linking the catalytic core to the stalk domains. By modulating the position and range of motion of the catalytic domain relative to the membrane, the hinge may help optimize substrate access within the crowded Golgi lumen.

Together, these findings demonstrate that hinge-region integrity modulates NAGPA activity in cells and that the full-length enzyme—including the C-terminal EGF-like stalk and membrane-tethered configuration—supports more efficient function than the catalytic core alone.

### Summary

In this study, we define the architecture, catalytic mechanism, and C-terminal stalk function of human NAGPA, the UCE required for generating the M6P lysosomal trafficking signal. Cryo-EM analyses reveal that NAGPA forms a noncovalent dimer composed of two catalytic cores and a C-terminal EGF-like stalk, resolving longstanding ambiguity in its oligomeric organization and extending prior structural work.

Mechanistically, comparison of apo and ligand-bound structures delineates the active-site environment and, together with prior biochemical and stereochemical evidence, supports a substrate-assisted S_N_i-like mechanism that explains α-retention without invoking a classic double-displacement pathway.

Functionally, our cell-based assay demonstrates that the C-terminal stalk actively enhances enzymatic performance: full-length, membrane-tethered NAGPA is more effective than the catalytic core, and perturbation of the hinge region leads to partial loss of activity.

In summary, these findings support a model in which the C-terminal EGF-like stalk positions the catalytic core for efficient engagement of glycan substrates in the membrane-proximal secretory environment, thereby directly coupling NAGPA architecture to its catalytic function.

## Experimental procedures

### Cloning and purification of recombinant human NAGPA

A human NAGPA construct was synthesized based on a previously reported design (11) and cloned into the pFastBac Dual expression vector (GenScript). The native signal peptide was replaced with melittin secretion signal to enable extracellular expression. The resulting construct (50–448 residues) comprising the catalytic domain and the CTD, was secreted in the culture media.

Recombinant bacmids were generated by transforming NAGPA plasmids into *E*. *coli* DH10Bac competent cells and subsequently used to transfect Sf9 cells for baculovirus production. Protein expression was carried out in Sf9 cells cultured in ESF921 medium (Expression Systems) following infection with 2% (v/v) recombinant baculovirus.

Secreted NAGPA was purified from the culture medium *via* its C-terminal Twin-Strep tag using Strep-Tactin affinity resin (IBA Lifesciences) in buffer containing 20 mM Hepes (pH 7.5) and 150 mM NaCl. Bound protein was eluted with 10 mM d-desthiobiotin or biotin. The eluate was further purified by size-exclusion chromatography on a Superdex 200 Increase 10/300 column (GE Healthcare) equilibrated in 20 mM Hepes (pH 7.5), 75 mM NaCl, and 75 mM KCl.

### Plasmid construction of hGAA-S1S3 and NAGPA

The hGAA–S1S3 plasmid was generated by inserting the S1S3 PTase gene downstream of the internal ribosome entry site using *Bsu36I* and *PmeI* restriction sites in a previously described vector (37). The human GAA coding sequence was cloned upstream of the internal ribosome entry site using *NheI* and *NotI* restriction sites.

The full-length WT human NAGPA construct in pcDNA3.1 was obtained from the Stuart Kornfeld laboratory (Washington University). NAGPA mutants were generated by site-directed mutagenesis using the QuikChange method (Agilent Technologies) and verified by whole-plasmid DNA sequencing.

### Generation of *NAGPA*^−/−^ ExpiCHO-S cells

ExpiCHO-S cells (Thermo Fisher Scientific, A29129) were cultured under standard conditions according to the manufacturer’s instructions. To generate a *NAGPA*^−/−^ cell line, the endogenous *NAGPA* gene was disrupted using CRISPR–Cas9 (GenScript, Z03393-100) with two guide RNAs targeting the coding region (sgRNA-1: 5′-GGTTTCTTCCGCATGAACAC-3′; sgRNA-2: 5′-GGGCTGCAGAACGCGCAGTT-3′).

Guide RNAs and Cas9 proteins were delivered into ExpiCHO cells by electroporation. Transfected cells were serially diluted and seeded into 96-well plates to isolate single clones. Individual clones were expanded and screened by PCR amplification of the targeted genomic region. Clones harboring deletions were further validated by Sanger sequencing.

### Cotransfection of hGAA–S1S3 and NAGPA

ExpiCHO-S *NAGPA*^−/−^ cells were cultured and transfected according to the manufacturer’s protocol. Briefly, 5 μg of hGAA–S1S3 plasmid was cotransfected with an equal amount of NAGPA variant plasmid or empty vector into 5 ml of cells at a density of 6 × 10^6^ cells/ml. Cells were incubated for 72 h at 37 °C.

Following expression, cells and conditioned media were separated by centrifugation at 700*g* for 15 min at 4 °C. The supernatant was transferred to a new tube and clarified by a second centrifugation at 16,900*g* for 15 min. Both conditioned media and cell pellets were frozen at −80 °C until further use.

### hGAA enzyme activity assay

hGAA activity in conditioned media was measured using a 4-methylumbelliferyl (4-MU) α-glucosidase assay. Samples were diluted 1:5 to 1:100 in GAA assay buffer (180 mM sodium acetate, 100 mM NaCl, 1.0 mg/ml bovine serum albumin, 0.02% Triton X-100, 0.02% NaN_3_, pH 4.8).

For each reaction, 25 μl of diluted sample was mixed with 25 μl of 1 mM 4-MU α-D-glucopyranoside (Sigma-Aldrich, M9766) in assay buffer and incubated at 37 °C for at least 30 min in duplicate. An eight-point 4-MU standard curve and a purified GAA positive control were included on each plate. Reactions were quenched by addition of 1 M glycine (pH 10.6).

Fluorescence was measured using a SpectraMax M2 plate reader (Molecular Devices) with excitation at 360 nm and emission at 460 nm. GAA activity (nmol/h/ml) was calculated using SoftMax Pro 7.1 software.

### CI-MPR affinity chromatography profiling of hGAA

To assess M6P-dependent binding, 100 μl of conditioned media was applied to a 1 ml in-house prepared column containing immobilized human CI-MPR agarose resin at a flow rate of 0.5 ml/min using an ÄKTA Purifier FPLC system (Cytiva) with Unicorn 7.6 software.

The column was washed with 5 ml of wash buffer (50 mM imidazole, 150 mM NaCl, 2 mM EDTA, 5 mM β-glycerophosphate, 0.05% Triton X-100, 0.02% NaN_3_, pH 6.8) to remove unbound proteins. Weakly bound species were eluted using a 5 ml linear gradient of 0 to 2 mM M6P in elution buffer (50 mM sodium acetate, 150 mM NaCl, 2 mM EDTA, 5 mM β-glycerophosphate, 0.05% Triton X-100, 0.02% NaN_3_, pH 4.8). Strongly bound species were subsequently eluted with 5 ml of elution buffer containing 10 mM M6P.

Fractions (0.2 ml) were collected in a 96-well plate, and hGAA activity in each fraction was quantified as described previously.

For analysis of GlcNAc-capped (covered) M6P species, 100 μl of the conditioned medium was preincubated with 6 μg of purified recombinant NAGPA for 1.5 h at 37 °C prior to chromatography, followed by the same separation procedure. Three biological replicates were carried out, and the ordinary one-way ANOVA was carried out using Prism to evaluate the statistical significance across samples.

### SDS–PAGE and Coomassie staining

Samples were briefly centrifuged and resolved on a 4 to 12% Bis-Tris SurePAGE gels (GenScript, M00653) using an XCell SureLock Mini-Cell system. Electrophoresis was performed in 1x Tris–MOPS–SDS running buffer at 150 V for 45 min. Gels were subsequently rinsed with deionized water and stained with InstantBlue Coomassie stain (Abcam, ab119211) for 30 min. After staining, gel was washed three times with deionized water and imaged using an Azure 400 imaging system under white light.

For conditioned medium, 10 μl of each sample was mixed with Pierce™ Lane Marker Reducing Sample Buffer (Thermo Fisher Scientific, 39,000) and boiled prior to loading. Relative protein band intensities were quantified using Fiji (ImageJ) (39).

### Western blot analysis of cell lysates

Cell pellets were lysed in radioimmunoprecipitation assay buffer (Pierce, 89901) supplemented with HALT protease inhibitor cocktail (Thermo Fisher Scientific, 78430), followed by centrifugation at 16,900*g* for 15 min (twice) to clarify lysates. Protein concentration was determined using a bicinchoninic acid assay (Thermo Fisher Scientific, 23227) according to the manufacturer’s instructions.

Equal amounts of protein (2.5 μg) were separated by SDS–PAGE as defined previously and transferred onto a 0.2 μm nitrocellulose membrane using a Trans-Blot Turbo system (Bio-Rad, 1704150). Membranes were washed three times with 1 × phosphate buffered Saline with tween-20 (PBST, VWR, 76371-736) and blocked in 5% nonfat milk in PBST for 1 h at RT with gentle agitation.

Membranes were incubated overnight at 4 °C with primary antibodies diluted in blocking buffer: rabbit anti-GAA (Abcam, ab137068, 1:5000), rabbit anti-NAGPA (Novus Biologicals, NBP2-83254, 1:1500), or rabbit anti-GAPDH (Thermo Fisher Scientific, PA1-987, 1:5000). After washing three times with PBST, membranes were incubated with HRP-conjugated goat anti-rabbit secondary antibody (Cell Signaling Technology, 7074, 1:10,000) for 1 h at RT.

Following three additional washes with PBST, signals were detected using SuperSignal West Femto substrate (Thermo Fisher Scientific, 34,094) and imaged. Band intensities were quantified using Fiji (ImageJ).

### Mass photometry

Mass photometry measurements were performed using 12 nM WT NAGPA on a Refeyn TwoMP mass photometer (Refeyn Ltd, Oxford). Instrument calibration was carried out using β-amylase (56 kDa monomer, 112 kDa dimer, 224 kDa tetramer) and thyroglobulin (660 kDa) standards to generate a mass calibration curve. Molecular mass distributions were obtained by fitting Gaussian functions to the detected particle peaks, from which the mean molecular mass and SD (σ) was determined.

### Cryo-EM grid preparation and data collection

Quantifoil R1.2/1.3 300-mesh gold grids (EMS, Q310AR1.3) were freshly glow-discharged prior to use. For apo NAGPA, 2.5 μl of purified protein at 0.3 mg/ml was applied to the grid. To mitigate preferred particle orientation, NAGPA was supplemented with 0.05% (w/v) n-octyl-β-D-glucoside immediately before applying on the grid.

Grids were vitrified using an FEI Vitrobot Mark IV operated at 95% humidity and 6 °C. Samples were blotted for 2.5 s using Whatman 595 filter paper at a blot force setting of 1, then plunge-frozen in liquid ethane cooled by liquid nitrogen. Frozen grids were stored in liquid nitrogen until imaging.

For the ligand-bound condition, NAGPA was incubated with 7 mM of GlcNAc-1-P for 1 h on ice prior to grid preparation, and vitrification was performed as described for the apo sample.

Cryo-EM data were collected on an FEI Titan Krios transmission electron microscope operating at 300 kV equipped with a Gatan K3 direct electron detector and controlled by SerialEM (40). Movies were recorded at a nominal magnification of 105,000 ×, corresponding to a calibrated pixel size of 0.828 Å. The defocus range was set from −1.1 to −1.6 μm. Each movie was dose-fractionated into 40 frames, with a total electron exposure of 58 e^−^/Å^2^.

### Cryo-EM data processing and 3D reconstruction

Cryo-EM data processing was performed in cryoSPARC v4.2 (41). Movie stacks were first subjected to patch motion correction, followed by contrast transfer function (CTF) estimation using patch CTF estimation. Micrographs were manually inspected, and those of poor quality were excluded from further analysis.

Initial particle picking was performed using the blob picker, followed by Topaz and template-based picking using templates generated from the initial dataset. Extracted particles were subjected to multiple rounds of 2D classification, *ab initio* 3D reconstruction, and heterogeneous refinement to remove contaminants and poorly aligned particles. Duplicates were removed prior to pooling particles for further processing.

The cleaned particle set was used for additional rounds of *ab initio* reconstruction and heterogeneous refinement. Particles belonging to high-quality classes were then refined using non-uniform refinement to generate a consensus map. Local refinement with a soft mask was applied to improve map quality in specific regions. C2 symmetry was imposed during refinement.

Subsequently, per-particle motion correction, CTF refinement, and reference-based motion correction were performed. To further improve map quality, iterative rounds of heterogeneous refinements were carried out using increasingly low-pass-filtered reference volumes to select the highest-quality particle subsets. The final reconstructions reached 2.84 Å resolution for the apo dataset and 3.10 Å for the GlcNAc-1-P dataset.

For the GlcNAc-1-P dataset, 3D-Flex analysis (42) was used to characterize conformational variability within the C-terminal EGF-like stalks. Additional particles selection based on 3D variability analysis further improve the final map to 3.01 Å resolution.

### Model building and refinement

An initial model of human NAGPA was generated using AlphaFold3 and docked into both unsharpened and EMReady2 (43)-sharpened cryo-EM maps. The model was manually adjusted in Coot v0.9.8.3 (44) and iteratively refined using real-space refinement in Phenix v1.20.1 (45).

The EGF1 domain was built *de novo* using CryoAtom (29) based on the apo map, whereas the remaining C-terminal domains were modeled using the AlphaFold3 prediction. The refined apo structure was subsequently used as the starting model for building the GlcNAc/phosphate-bound complex.

Final models were validated using MolProbity (46). Figures were generated using UCSF ChimeraX v1.6 (47), and two-dimensional schematics of the dimer interface and protein–ligand interactions were generated with LigPlot+ (48). Chemical structures and reaction schemes were prepared using MarvinSketch (ChemAxon).

### Sequence alignment of NAGPA homologs and related phosphodiester glycosidases

Protein sequences of human NAGPA homologs and related phosphodiester glycosidases were retrieved from the UniProt database (49). Representative sequences were selected from eight species: *Homo sapiens* (NAGPA_Human), *Cavia porcellus* (H0VTT5), *Gallus gallus* (A0A8V0Z8N9), *Xenopus laevis* (A0A974DM62), *Danio rerio* (F1QSF9), *Oryzias latipes* (H2MKM3), *Bacteroides ovatus* (A0A1G6G3R0), and *Mycobacterium tuberculosis* (A0A045I364).

Among these, the *G*. *gallus* and *O*. *latipes* proteins are annotated as NAGPA, whereas the *M*. *tuberculosis* protein is annotated as a phosphodiester glycosidase family protein. Multiple-sequence alignments were generated using Clustal Omega (50) with the default parameters and visualized using ESPript 3.2 (51).

### Data availability

The cryo-EM maps and corresponding atomic models of human NAGPA have been deposited into the Electron Microscopy Data Bank and Protein Data Bank under accession codes EMD-76186 and 11YG for the apo state, and EMD-76185 and 11YF for the GlcNAc/phosphate-bound state. All other data supporting the findings of this study are contained within the article and Supporting Information.

## Supporting information

This article contains Supporting information, including cryo-EM processing workflows, reconstruction statistics, sequence alignments, and additional cell-based assay data.

## Conflict of interest

R. M. and L. L. are employees and shareholders of M6P Therapeutics. All other authors declare that they have no conflicts of interest with the contents of this article.
