# Supplementary material for: Structure determination reveals the mechanistic basis of mannose-6-P signal generation by the dimeric lysosomal uncovering enzyme NAGPA
Source: J Biol Chem. 2026 Jul 13;302(9):113329. doi: 10.1016/j.jbc.2026.113329 (PMC13474241; doi:10.1016/j.jbc.2026.113329)
Supplement: Supplementary file 1 [file mmc1.docx]

Supplementary information for

**Structure Determination Reveals the Mechanistic Basis of Mannose-6-P Signal Generation by the Dimeric Lysosomal Uncovering Enzyme NAGPA**

*by Farha Khan et al.*

This document contains

One supplementary table

Seven supplementary figures

**Supplementary Table 1. Cryo-EM data collection, reconstruction, model refinement, and validation statistics.**

|  | Apo NAGPA | NAGPA + GlcNAc-1-P |
| --- | --- | --- |
| **Data collection and processing** |  |  |
| Microscope | FEI Titan Krios | FEI Titan Krios |
| Voltage (kV) | 300 | 300 |
| Detector | K3 | K3 |
| Operation mode | CDS | CDS |
| Total electron exposure  on sample (e^-^/Å^2^) | 58 | 58 |
| Number of movie frames | 40 | 40 |
| Magnification | 105K | 105K |
| Pixel size (Å) | 0.828 | 0.828 |
| Targeted undefocus range (μm) | 1.1–1.6 | 1.1 – 1.6 |
| Number of collected movies | 16,328 | 18,859 |
| Symmetry imposed | C2 | C2 |
| Final particle images (no.) | 184,476 | 372,868 |
| Map resolution (Å)  FSC=0.143 | 2.84 | 3.01 |
| **EMDB ID** | EMD-76186 | EMD-76185 |
| **PDB ID** | 11YG | 11YF |
| **Model composition**  Non-hydrogen atoms  Protein residues  Glycans (molecules)  Ligands (molecules) | 5,600  722  (10)  - | 5,640  722  (10)  (4) |
| **B-factors (Å^2^)**  Protein  Glycans  Ligands | 72  126 | 78  166  124 |
| **R.m.s. deviations**  Bond lengths (Å)  Bond angles (°) | 0.004  0.732 | 0.009  0.928 |
| **Validation**  MolProbity score  Clashscore  Poor rotamers (%) | 1.52  3.70  0.00 | 1.95  10.12  0.00 |
| **Ramachandran plot**  Favored (%)  Allowed (%)  Disallowed (%) | 94.85  5.15  0.00 | 93.45  6.55  0.00 |

**Supplementary Figure 1. Cryo-EM data processing for the apo NAGPA dataset.** After standard pre-processing, extracted particles were subjected to multiple rounds of 2D classification, *ab initio* 3D reconstruction, and heterogeneous refinement to remove contaminants and poorly aligned particles. With a cleaned up 436,681 particle stack, per-particle motion correction, CTF refinement, and reference-based motion correction were performed to achieve a 3.18 Å refinement in C1. To further improve map quality, five rounds of heterogeneous refinements were carried out using increasingly low-pass-filtered reference volumes to further select the particles. The final reconstruction with 184,476 particles reached 2.84 Å resolution in C2 symmetry.

**Supplementary Figure 2. Cryo-EM data processing for the NAGPA dataset complexed with GlcNAc-1-phosphate.** After standard pre-processing, extracted particles were subjected to multiple rounds of 2D classification, *ab initio* 3D reconstruction, and heterogeneous refinement to remove contaminants and poorly aligned particles. With a cleaned up 1,039,345 particle stack, per-particle motion correction, CTF refinement, and reference-based motion correction were performed to achieve a 3.35 Å refinement in C1. To further improve map quality, two rounds of heterogeneous refinements were carried out using increasingly low-pass-filtered reference volumes to further select particles. After another round of reference-based motion correction, an improved resolution of 3.07 Å was achieved with 515,857 particles in C1. 3D-Flex analysis reveals significant flexing motion of the hinge domains. Another round of particle selection with 3D variability analysis resulted in a final reconstruction of 3.01 Å in C2 symmetry.

**Supplementary Figure 3. Overview of the final cryo-EM reconstructions for the apo** (**A**) **and GlcNAc/phosphate-bound** (**B**) **datasets**. In each panel, the local resolution map, Fourier shell correlation (FSC) curve, and particle orientation distribution are shown.

**Supplementary Figure 4. Comparison of CryoAtom and AlphaFold3-based models of human NAGPA**. Structural overlay of the CryoAtom *ab initio* model (green) and the final refined model seeded from the AlphaFold3 (AF3) prediction (gray). The two models are in close agreement across the catalytic domain and remain largely consistent through the first EGF-like domain up to approximately His350, beyond which the CryoAtom model diverges from the refined structure.

**Supplementary Figure 5. Molecular basis for the ligand recognition in NAGPA. (A)** Stereo view of the GlcNAc and phosphate binding in human NAGPA. Key residues involved in the ligand binding are shown in stick representation. **(B)** Sequence alignment of catalytic domains from phosphodiester glycosidases homologous to human NAGPA. UniProt IDs of the aligned sequences are *Homo sapiens* (Q9UK23), *Cavia porcellus* (H0VTT5), *Gallus gallus* (A0A8V0Z8N9), *Xenopus laevis* (A0A974DM62), *Danio rerio* (F1QSF9), *Oryzias latipes* (H2MKM3), *Bacteroides ovatus* (A0A1G6G3R0), and *Mycobacterium tuberculosis* (A0A045I364). Conserved residues are highlighted in red, and secondary structure elements from the human enzyme are mapped above the alignment. Disulfide-bonded cysteines were labeled with paired blue letters (b–e). Residues involved in GlcNAc and phosphate binding are marked with red arrowheads.

**Supplementary Figure 6. Expression and functional analysis of hGAA co-expressed with different NAGPA constructs. (A)** SDS–PAGE analysis of conditioned media from transfected CHO cells showing broadly similar secretion profiles across samples. Recombinant hGAA migrates at ~130 kDa. In sample 5016, an additional lower-molecular-weight band is observed, likely corresponding to the secreted soluble NAGPA catalytic domain. **(B)** Quantification and statistical analysis of M6P uncovering efficiency, expressed as the percentage of strongly CI-MPR-bound hGAA (elution with 10 mM M6P) relative to total recovered activity for each sample. The catalytic-domain construct showed significantly reduced activity relative to wild-type NAGPA, whereas only the quadruple mutant at the hinge region significantly affected activity. Data from three independent biological replicates are plotted as individual points overlaid on each bar. Statistical significance is indicated as p ≤ 0.01 (**) and p ≤ 0.0001 (****); “ns” denotes not significant.

**Supplementary Figure 7. Reproducibility of cell-based assay across independent expression batches.** Each panel shows results from one batch: **(A)** batch 1, **(B)** batch 2, and **(C)** batch 3. Recombinant GAA was analyzed alone or after incubation with human UCE and NAGPA constructs, including wild-type UCE, the catalytic mutant **D286A**, the hinge region double mutant **R328A/H325A**, the **R102A/H344A** mutant, the combined **R102A/H344A/R328A/H325A** mutant, and the isolated catalytic-domain construct. The percentage values indicate the fraction of recovered GAA activity within the indicated elution regions.
